# Weekly and monthly patterns in suicide-related emergency care visits

**DOI:** 10.1192/j.eurpsy.2021.1877

**Published:** 2021-08-13

**Authors:** D. Hernández-Calle, M.F. Bravo-Ortiz

**Affiliations:** Psychiatry, Hospital Universitario La Paz, Madrid, Spain

**Keywords:** Help seeking, Suicide

## Abstract

**Introduction:**

Suicidal seeking help seeking behaviour has with associated with seasonal pattern that reflect an array of psychosocial factors. Its understanding is paramount for improving psychiatric emergency care.

**Objectives:**

Our aim was to analyse weekly and monthly seasonality on emergency department visits due to autolytic phenomena

**Methods:**

Daily urgency visits from suicidal phenomena (including suicide attempt and ideation) were extracted from electronic medical records of Hospital Universitario La Paz from 1st January 2019 to 31st December 2019. A poisson multivariate model was performed with day of the week and month as covariates. Predictive margins were estimated

**Results:**

Psychiatric emergency visits due to suicidal phenomena were less frequent in Saturday and Sunday (1.8 visits per day) than weekdays (2.5 visits per day). Peaks were observed in February and September, being April and May the months with fewer visits

**Conclusions:**

A weekly season pattern was observed with less psychiatric emergency visits due to suicidal phenomena during weekends. They picked during colder months and were less frequent during spring time

**Disclosure:**

No significant relationships.

